# Parents' Perspectives on a Computer Game–Assisted Rehabilitation Program for Manual Dexterity in Children With Cerebral Palsy: Qualitative Analysis of Expectations, Child Engagement, and Benefits

**DOI:** 10.2196/24337

**Published:** 2021-05-31

**Authors:** Anuprita Kanitkar, Sanjay Tejraj Parmar, Tony J Szturm, Gayle Restall, Gina Rempel, Nariman Sepehri

**Affiliations:** 1 University of Manitoba Winnipeg, MB Canada; 2 SDM College of Physiotherapy SDM University Dharwad India

**Keywords:** cerebral palsy, parents' expectations, fine motor function, object manipulation, computer game–based treatment protocol, parents, motor function, computer games, rehabilitation, game-based rehabilitation, gross movement, children

## Abstract

**Background:**

Children with motor impairments affecting the upper extremity benefit from task-specific therapy, such as constraint-induced movement therapy. However, there is a need to improve engagement and compliance with task-specific exercise programs that target manual dexterity for children with cerebral palsy (CP). A computer game–based rehabilitation (GRP) platform was developed that combines fine manipulation and gross movement exercises with engaging game activities appropriate for young children with CP.

**Objective:**

The objectives of this qualitative analysis were to compare parents’ perspectives and opinions about expectations, challenges, and benefits between 2 interventions.

**Methods:**

A mixed methods, randomized controlled trial (RCT) was conducted to examine the feasibility and estimate the effect size of 2 exercise programs for rehabilitation of manual dexterity of children with CP using either GRP or conventional therapy. Parents of 26 of the children who completed the GRP program (n=33) and parents of 15 of the children who completed the conventional therapy program (n=27) participated in the interviews. A general conductive approach was used to analyze the data recorded during the parents’ interviews.

**Results:**

Five themes captured the range of the parent’s experiences, viewpoints, and ideas: (1) parents’ expectations, (2) child’s engagement with therapy, (3) positive effects of the interventions, (4) challenges, and (5) improving the protocol.

**Conclusions:**

Parents from both groups recognized that their expectations related to improving children’s object handling and manipulation skills including participation in activities of daily life were addressed during the 16-week therapy program. Parents perceived a change in the children’s level of independence in their daily tasks at home, school, and leisure activities.

**Trial Registration:**

ClinicalTrials.gov NCT02728375; https://clinicaltrials.gov/ct2/show/NCT02728375

## Introduction

Children with motor impairments of the upper extremity due to cerebral palsy (CP) face numerous difficulties in their activities of daily living (ADL), in participation in school, and during play. Task-specific rehabilitation programs such as Constraint Induced Movement Therapy (CIMT) [1] and hand-arm bimanual intensive training (HABIT) [2] have shown positive results when provided by therapists in a one-on-one clinical setting with high repetitions of task practice [3].

Recent studies have introduced digital media to enhance the play-based therapy protocols for children with CP [4]. These include the Wii [5] and Kinect [6] commercial gaming systems and custom gaming systems that use robotic manipulanda [7] or sensor-equipped gloves [8]. These gaming systems can detect arm segment motion or finger motion in the case of the instrumented glove. These sensor motion signals are used to interact with virtual objects or to control a game paddle for play; however, these cannot be used to couple goal-directed object handling and manipulation exercises with computer games. In addition, these gaming systems come with a limited number of games suitable for young children with motor impairments, whereas there is a large number of inexpensive and readily available common and modern commercial games that are engaging and can be played with a computer mouse or equivalent.

Using game-assisted rehabilitation technologies is still a relatively new discipline. There is a need to develop study designs to explore the implementation, acceptability, and appropriateness of these technology-based interventions [4-15]. Based on this information, a computer game–based rehabilitation platform (GRP) was developed [9-11] to focus on object handling and manipulation tasks. The GRP uses a miniature commercial wireless inertial based (IB) computer mouse, which links physical movements with engaging, interactive computer games. The precision and responsiveness of the IB mouse are equivalent to that of a standard optical computer mouse. When the IB mouse is attached to an “exercise” object, the manipulation of the object is used to control the motion of a computer cursor or game paddle. Importantly, the IB mouse can be attached to a broad range of objects with different physical properties and functional demands. Therefore, many objects of varied size, shape, weight, and surface properties can be used in the game-assisted exercise program. Several principles of motor learning are incorporated in the GRP [12-14], including task-specific training of object handling and manipulation, multisensory stimulation, and feedback or knowledge of performance.

A mixed methods, exploratory, randomized controlled trial (RCT) was conducted to explore parental views of children's experiences with their respective exercise programs and to provide an estimate of the treatment effect size that would direct a future full-scale RCT.

Qualitative analysis is important to gain knowledge from parents' experiences with the GRP program and to reinforce and strengthen the evidence obtained from a quantitative analysis of treatment effects [16]. It is necessary to explore whether the children's goals were met. Children’s experiences and beliefs can directly influence engagement in the intervention [17]. The results of the qualitative analysis are presented in this paper, while the quantitative findings will be reported in a separate paper. The objectives of the present study were to investigate parental views of children's experiences about expectations and benefits of the GRP exercise programs targeting the hand and arm function of young children with CP, expectations and benefits of the conventional therapy programs targeting the hand and arm function of young children with CP, engagement with the therapy, positive effects of the interventions, and challenges with implementing the exercise program.

## Methods

Children diagnosed with CP, aged 4-10 years, who were scheduled to receive therapy and met the following inclusion criteria were recruited: Gross Motor Function Classification System (GMFCS) levels 1-3 [18], manual Ability Classification System (MACS) levels 1-3 [19], level of spasticity on the Modified Ashworth Scale (MASH) from grade 1 to 1+ [20], score ≥17 on the pediatric version of the Mini-Mental State Evaluation (MMSE) [21].

After the initial screening process, parents provided written informed consent.

The initial session included the following assessments: Grasp and Visual-Motor Integration (VMI) subtests of the Fine Motor Quotient of the Peabody Developmental Motor Scale-2 (PDMS-2) [22] and a computer game–based assessment. A miniature wireless IB computer mouse (Scoop Pointer Remote Model RXR1000-0302E, Hillcrest Lab, Rockville, MD) was secured using Velcro to 5 test objects chosen for the assessment of manual dexterity. Performance measures for the 5 object manipulation tasks included the success rate, response time, and movement error developed [9-11].

In the experimental group (XG) intervention, the initial exercise protocol for each participant was established based on the child’s and parents' goals, level of impairment, and functional status. A typical session for the XG consisted of a computer game–assisted exercise program.

**Figure 1:**
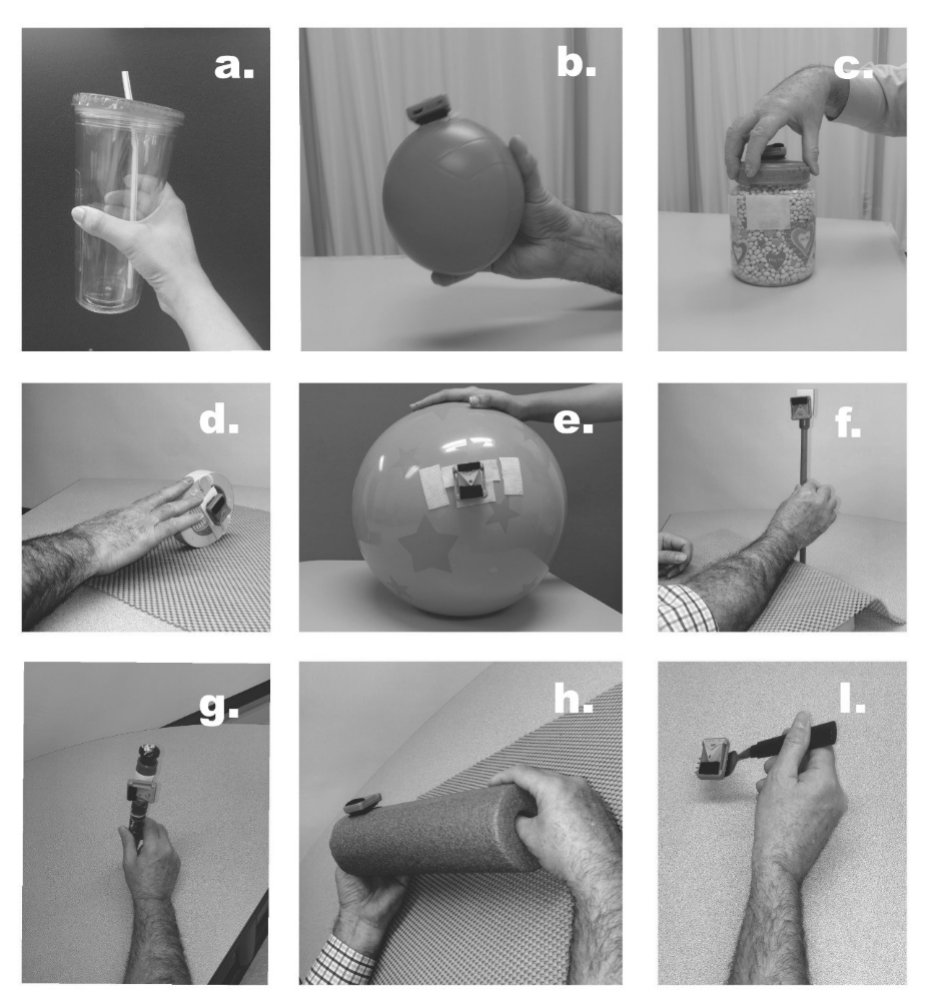
Example object manipulation tasks illustrated by a therapist: (A) handling a plastic cup with wrist radioulnar deviation while a midprone forearm is maintained with elbow resting on a surface; (B) handling a sponge ball (12-cm diameter) using wrist flexion- extension while maintaining a palmar grasp and midprone forearm with elbow resting on a surface; (C) rotation of a jar lid (opening and closing) using a 5-finger grasp over the lid and wrist radioulnar deviation and 5-finger flexion-extension with elbow resting on an elevated surface; (D) rolling movement of a toy wheel, manipulated using the index and middle fingertips and moved using shoulder flexion-extension; (E) bimanual beach ball manipulation using open hands and elbow flexion-extension for movement while forearms are maintained in a midprone position and shoulders maintained in mild flexion; (F) rolling a stick using index and middle fingertips and thumb opposition, while maintaining a neutral wrist and midprone forearm with elbow resting on an elevated surface; (G) manipulating a pen with index and middle fingers and thumb opposition using a tripod grip and maintaining a neutral wrist resting on the table; (H) bimanual roll of a pool noodle using thumbs and finger flexion-extension and fingertips for manipulation while a midprone forearm is maintained with elbow resting on a surface; (I) fork manipulation using a tip-to-pad grip with the index and middle finger, thumb opposition, and forearm supination-pronation with elbow resting on an elevated surface.

Children were provided between 6 and 8 IB mouse–equipped objects for exercise, which were used to play several computer games. The objects were everyday items such as balls of various sizes; daily objects like spoons, glasses, and cups; school-related objects such as markers; and play-based objects such as toys. These objects represented a wide range of physical properties requiring different modes of manipulation and functional demands. The GRP can take advantage of ergonomic properties of common objects to amplify limited and small voluntary movements during gameplay and then allows opportunities to use exercise objects with more challenging demands. Figure 1 presents a description of several object manipulation tasks used in this study for game-assisted repetitive task practice (RTP). 

Treating physiotherapists instructed children how to perform the various tasks with the desired hand and arm segment motions and to avoid substitution with associated movements. Computer games that best suited the object manipulation tasks were chosen for the exercise program. The games were chosen based on the following game properties: (1) movement amplitude required to move the game paddle, (2) game speed, and (3) game precision requirement. Many inexpensive arcade-style computer games are readily available online and can be downloaded from websites such as Big Fish games [23]. Multimedia Appendix 1 presents a list of computer games used and a description of movement and cognitive requirements. This protocol was updated every 4 weeks based on the child's improvements, current goals, and functional demands.

The protocol was updated on week 4, week 8, and week 12. The intervention was performed for 16 weeks. For example, certain features of the object manipulation tasks and variables of the computer games were adjusted, as tolerated, to increase the challenge and progress the exercise program. Of note, objects with different sizes, shapes, weights, and surface frictions were used to increase the physical demands of the tasks. Surface friction was adjusted using various materials such as kitchen drawer liner material, various rubberized materials, as well as plastic and Styrofoam objects versus objects with leather coverings. Game speed and then movement precision (size of target objects and game paddle) were increased (ie, the speed-accuracy relationship). Movement amplitude was increased by adjusting the mouse sensitivity. In addition, cognitive load was increased by selecting games with an increasing number of distractor objects (ie, dual-task interference). Of note, most children were competitive and became frustrated if they were not successful in game play. Therefore, game play success was usually set to 60% or higher for all combinations of objects, game settings, and game types.

In the control group (CG) intervention, we used a comprehensive physical therapy protocol based on the goal-oriented, repetitive task practice–based principles of modified CIMT and HABIT. Therapy protocols were individualized for every participant according to their level of impairment and pre-set goals. A variety of arm and hand activities were practiced, such as reaching for rings, ball throwing, clay activities, picking marbles from sand, and putting pellets and pegs into sockets. These tasks were practiced by the child with the guidance and assistance of a therapist.

Both the XG and CG protocols were designed and updated based on the recommendations of RTP-based protocols such as CIMT [24,25]. The recommended period for such protocols is 3-4 weeks in order to see improvement in functional goals [24-26].

For the qualitative data collection, parents of children in the XG and CG were invited to participate in interviews conducted at the end of the intervention. The purpose of the interviews was to understand parental views of children's experiences with the interventions. An interviewer who was blinded to the intervention received by the parent's child conducted all the interviews using a semistructured interview guide (Textbox 1). Interviews were conducted in local languages or parents' preferred language. Parents were encouraged to describe and explain their ideas, thoughts, and opinions. The interviewer noted any nonverbal communications and other observations in field notes. The interviews were audio recorded. Audio recordings were both professionally transcribed and translated to English.

**The interview guide.**

1. When you agreed to participate, how did you hope your child would benefit from the therapy program?
2. What did you like about the therapy program?
3. What was difficult or challenging about implementing the therapy program for your child and you?
4. What did you think about the computer games/exercises your child was asked to play?
5. How did your child respond to the games/exercises? Were there games/exercises which your child did not seem to enjoy?
6. How did technology integrate into your daily life?
7. Would you want your child to continue with the same type of therapy program? Why or why not?
8. Any other suggestions?

The analytical framework of interpretive description was used for thematic interpretation [27]. Translated transcripts and the field notes were initially read by one researcher who developed the coding system by paraphrasing, generalizing, and abstracting the written transcripts of each interview. A second researcher scrutinized the coded data and identified any additional unique responses and codes. The 2 researchers then met via video calls to compare their analyses and resolve disagreements in a final code system. 

These coded responses and direct quotes from the interviews were back-translated to the parents’ preferred languages. Parents were asked to review this material and provide feedback about the accuracy of the researchers’ interpretations. This was done as a member-checking procedure to promote trustworthiness and fidelity [28]. After receiving parent feedback, the data and coding were again reviewed by both researchers and organized into final themes and subthemes described in the Results section.

## Results

Table 1 presents the demographic and clinical data by group (XG: n=33, mean age 7.2 years; CG: n=30, mean age 7.8 years). There were no significant differences between groups at baseline in age, MMSE, or PDMS-2 Grasp/VMI scores. The majority of children were at GMFCS levels I-III and MACS levels I-III. Both groups had 6 children at a level I.

**Table 1:** Demographic and clinical characteristics of the groups.

Characteristics	Control group (CG; n =30)	Experimental group (XG; n=33)	*P* value^a^
Age (years), mean (SD)	7.8 (1.9)	7.3 (2.1)	.20
MMSE^b^, mean (SD)	27.7 (1.4)	29.0 (0.3)	.40
GMFCS^c^	I, n=6; II, n=14; III, n=7	I, n=8; II, n=15; III, n=10	N/A^d^
MACS^e^	I, n=6; II, n=16; III, n=11	I, n=4; II, n=15; III, n=8	N/A^d^
PDMS-2^f^ Grasp, mean (SD)	38.8 (4.2)	38.5 (3.1)	.80
PDMS-2 VMI^g^, mean (SD)	110.4 (10.1)	107.6 (8)	.50

^a^*t* test results.

^b^MMSE: Mini-Mental State Examination.

^c^GMFCS: Gross Motor Function Classification System.

^d^N/A: not applicable because *t* tests were not performed for comparisons.

^e^MACS: Manual Ability Classification System.

^f^PDMS-2: Peabody Developmental Motor Scales - Second Edition.

^g^VMI: Visual-Motor Integration.

Three participants from the CG withdrew from the study due to a change of school, the commute, and transportation-related issues (see the CONSORT diagram in Multimedia Appendix 2). Parents of 26 of the children from XG and parents of 15 of the children from the CG agreed to participate in the interviews. However, after the member-checking procedure, only 24 parents from the XG and 14 parents from the CG responded in person or via phone in addition to their written feedback.

The following 5 themes and subthemes captured the range of parent's experiences, viewpoints, and ideas: parents’ expectations, use of computers, child’s engagement with therapy, positive effects of the interventions, challenges, and improving the protocol. Examples of the parents' direct quotes for each theme are provided. 

### Parents' Expectations

All participants had been undergoing conventional therapy for at least 2 years, and their reported reasons for joining the study were varied. Most parents expressed their willingness to join the trial because therapy would focus on manual dexterity (Table 2, quotes 1 and 2). Many parents from both groups (XG, n=22; CG, n = 11) expressed concerns regarding a gap in current therapy services that often focus on arm movements and not on manual dexterity (Table 2, quote 1). Many parents from both groups (XG, n=13; CG, n=5) expressed their concerns regarding their child's inability to participate in both play and school activities due to lack of hand-eye coordination (Table 2, quotes 3-6). Considering that the protocol required children to focus on the computer screen while performing fine motor tasks, some parents assigned to the GRP program believed that this therapy might improve their children's hand-eye coordination as well as their attention span (Table 2, quote 4). Eight parents made the decision to join the study with the hopes the intervention would improve their child's handwriting (Table 2, quote 4).

**Table 2:** Parents’ expectations when joining the program.

Group, parent, and ID		Quote number	Example quote
**XG^a^**			
	Mother 12	1	“My daughter had hand and leg weakness for a few years. We have tried many places; they worked with her hand for picking up toys and playing with putty and elastics, but she is still not able to use her hand independently and normally. The therapy is just not working so far, so we decided to come here to SDM. Then, (the therapist) told us about this new study. I approached this treatment because I hoped she will practice activities using different objects with her hands, and with time and practice, those actions will improve.”
	Mother 4	2	“My child had problems with fine finger movements. Our consultant physician had told us that he was never going to use his fingers. When we heard about this program, we thought that this program might help.”
	Mother 5	3	“My daughter had difficulty in moving her right hand, that was the main thing, but we are hoping that this (CRP^b^ protocol) will also help her in analyzing things and improve her concentration.”
	Father 23	4	“We joined this therapy with the hopes that it will improve her handwriting along with hand-eye coordination.”
	Mother 15	5	“When we started this therapy, we hoped this therapy with computers will increase his interest and attention in studying.”
	Mother 18	6	“Knowledge of technology is always helpful as today's life is full of technology; it would help him in concentration, overall development using technology, and hand movement and motivate him to play and learn.”
	Mother 22	7	“It is very useful to communicate and relate to the world. In this way, it helps my child to learn and use a computer through this therapy.”

^a^XG: experimental group.

^b^CRP: computer games–based rehabilitation protocol group.

### Use of Computers

The use of computers for participants from a developing country, such as India, was a novelty. Most participants and many of their parents do not have access to computers and electronic devices. Many parents joined the GRP protocol with the goal of getting their child acquainted with computers (Table 3, quotes 1-3). In addition, many parents expressed that technology would play a major role in helping children achieve future goals such as employment and university-level education (Table 3, quote 1). Most parents from the XG reported that their child had never interacted with computers before (Table 3, quotes 1 and 2). Most parents presented an overall positive attitude towards the use of computer games and allowing their children to play computer games as part of therapy (Table 3, quote 1).

**Table 3:** Parents’ responses about the use of computers.

Group, parent, and ID			Quote number		Example quote
**XG^a^**					
	Mother 15	1		“When we started this therapy, we hoped this therapy with computers will increase his interest and attention in studying. It will help him in his future, when he goes to college or work.”	
	Mother 18	2		“Computers seem so attractive, and he wants to learn how to use them. Knowledge of technology is always helpful as today's life is full of technology; it would help him in concentration and overall development using technology. We hope he improves his hand movement, and it motivates him to play and learn along with his classmates.”	
	Mother 22	3		“It (technology) is very useful to communicate and relate to the world. In this way, it helps my child to learn and use a computer through this therapy. I think it will be more fun also, which means less complaints.”	
	Father 23	4		“From this treatment, she acquires the knowledge of computers and also gets to know other information in technology.”	

^a^XG: experimental group.

Many parents believed that introducing their children to computers while performing play-based therapy would create positive learning experiences (Table 3, quote 3). Parents expressed their intentions to join the therapy and later continue the therapy so that their child's communication skills could improve by boosting their confidence while also improving their social interactions in schools and later in life (Table 3, quotes 3 and 4). Parents expressed that basic knowledge of computers and getting used to using computers will help children because it is useful to communicate and relate to today's world of technology (Table 3, quotes 3 and 4).

### Child's Engagement With Therapy

Many parents expressed the view that, as the children get older, conventional therapy becomes repetitive and boring. In the XG, 19 parents commented that it was easier to convince children to perform exercises using the GRP than conventional exercises (ie, based on prior therapy; Table 4, quotes 1 and 2). Many parents perceived that their child found most of the chosen computer games to be engaging and viewed the exercises as play (Table 4, quotes 3-5). From the CG, 7 parents commented on a lack of interest in their child in participation during the therapy session (Table 4, quotes 6-9). Often, parents observed improvements in their children during the initial sessions, but the children lost interest with time (Table 4, quotes 6-8).

**Table 4:** Parents’ responses about their child’s engagement with therapy.

Group, parent, and ID		Quote number	Example quotes
**XG^a^**			
	Mother 12	2	“Earlier during (conventional) therapy sessions, my child used to get frustrated and annoyed quite easily. Then, we started the computer games therapy, and now my child feels relaxed and enjoys these therapy tasks while playing computer games. Because of this, we have observed a lot of progress in her behavior; it’s positive.”
	Mother 24	3	“She does really well in the game. She likes to play the fish game because of the variety of fish there in that game where one fish attacks and eats all the other fish. So, by this, the memory power is increased.”
	Mother 57	4	“In this treatment, my son liked all the therapy games. He learned to play games using various objects. His grip has become stronger now, and the main thing is that he is liking therapy now.”
	Mother 47	5	“Maybe the kids would enjoy this more than conventional therapy. He was bored with conventional therapy; now, he is coming more easily for computer games (for CRP^b^-based therapy) than conventional therapy.”
**CG^c^**			
	Parent 1	1	“Kids nowadays do not like traditional therapy. My child gets annoyed and bored easily there.”
	Mother 26	6	“She is a bit tired after all this time, but she was giving good responses at first; she has improved a lot”
	Father 10	7	“We have been doing therapy for almost 10 years now; he is very bored of therapy, and he gets angry and cranky now.”
	Mother 34	8	“Well, as she is growing up, she is certainly developing moods, so the therapy needs to be made more interesting for her”
	Mother 2	9	“Uhh, (child's name) is still small, I hope that when she grows up, she gets a little bit more motivated to do this. This is for her own good.”
	Mother 56	10	“She is doing well. She likes to play with the ball and other fun things.”

^a^XG: experimental group.

^b^CRP: computer games–based rehabilitation protocol group.

^c^CG: control group.

### Positive Effects of the Interventions

From the XG, 22 parents reported that they perceived improvements in their children's manual dexterity, object manipulation skills, and hand-eye coordination (Table 5, quotes 1-4). Some parents reported that their children improved not only in their “ability to pick up and hold objects” but also in their ability to “manipulate objects with more precision” and “stability” in unimanual as well as bimanual activities (Table 5, quotes 1 and 2). Improvements were also observed by parents from the XG in using technology-based gadgets like phones and laptops due to improved confidence levels (Table 5, quotes 3 and 4).

**Table 5:** Parents’ responses about the positive effects of the interventions.

Group, parent, and ID		Quote number	Example quotes
**XG^a^**			
	Mother 25	1	“I’m happy to see my daughter using both hands to hold toys (objects) and playing games. I also assume that she tries to catch the game toy (practice object for therapy) and play the (computer) game. So, I think now she knows how to move her hands (using therapy objects) while playing the (computer) game.”
	Mother 42	2	“He learnt to play games holding various objects. I can see him using his hands more now when eating and playing.”
	Mother 38	3	“With this game therapy, he is able to play and understand other game concepts even when he is playing in the apartment with friends.”
	Father 33	4	“In our day-to-day life, we hardly have any need for technology-based things. Since this therapy program has started, we have observed drastic changes in my child’s day-to-day life. My child’s handwriting is improved.”
	Mother 22	5	“Yes, he seems smarter, he knows about colors, and he knows about shape and directions, He sits back properly, His hand fingers are more active, and he also gets some exercise for the eyes.”
	Mother 4	7	“He has done well so far; he is more independent. This therapy program helped him with that.”
**CG^b^**			
	Mother 7	6	“Dr. (therapist’s name) is simply the best! We can already see that he is using his right hand for more activities; he pays more attention.”
	Mother 26	8	“I like that the sessions are one-on-one and that the therapists look after her alone for the whole time. You can see the difference in her. She is not irritated and angry like she used to be all the time.”

^a^XG: experimental group.

^b^CG: control group.

Some parents commented that they observed improvements in the quality of arm movements as well as posture and balance while sitting and playing computer games (Table 5, quotes 5 and 6). Many parents commented about their perception that the use of computer games had a positive impact on their child's cognitive abilities (Table 5, quote 5), hyperactivity (Table 5, quote 5), reduced attention span (Table 5, quote 6), and anger (Table 5, quote 8). One set of parents mentioned that their 4-year-old child developed better color and pattern recognition in addition to spatial orientation (Table 5, quote 6).

Most parents in the CG also provided positive feedback. Parents appreciated the one-on-one therapy sessions (Table 5, quotes 7 and 8). Parents gave positive feedback using words such as “improved independence,” “good results,” “happy,” and “thankful for therapy” (Table 5, quote 7). Many parents in the CG reported improvements in the child's upper extremity function and increased level of independence (Table 5, quote 8). Parents from the CG observed that the children were actively performing daily tasks such as self-feeding and dressing activities since their participation in the GRP protocol.

### Challenges

The experimental GRP protocol was updated every 4 weeks. Four parents felt that the 4 weeks was too long a period between exercise or game updates and commented that their child lost interest with their exercise when a game was used over the 4-week time period (Table 6, quotes 1 and 2). Parents expressed that it was challenging to understand the protocol during the first couple of sessions and 2-4 sessions were required for the child to learn how to use the gaming system (Table 6, quote 5).

The most common challenges reported by parents from the CG were about their child's compliance with therapy and engagement or interest with the exercise program (Table 6, quotes 3 and 4). 

**Table 6:** Challenges faced by parents.

Group, parent, and ID			Quote number		Example quotes
**XG^a^**					
	Mother 57	1		“We didn’t have any difficulties during computer therapy, but I would have liked to see him do more games. Once he has achieved one game, see to it that you please give him other challenging games that will help him to improve more.”	
	Mother 57	2		“When we started this therapy, our hope was that this will help our son in learning computers and his hand and arm will get stronger, more skillful.”	
	Mother 18	5		“First 3-4 sessions, he struggled. It took time to realize what he was supposed to do, but now after so many sessions, he enjoys it.”	
**CG^b^**					
	Mother 1	3		“Sometimes my child doesn’t like the objects because he finds it a little difficult to hold and move the object.”	
	Mother 10	4		“Well, the session takes really long; it’s time consuming, I wish we could reduce the duration of the activities. He is starting to get tired of the things to do on the table.”	

^a^XG: experimental group.

^b^CG: control group.

### Improving the Protocol

Many parents suggested adding educational games such as math and language games as well as computer games with a broader range of cognitive content (Table 7, quote 1). One parent from the CG suggested that the activities should be changed more regularly and to incorporate play-based, child-parent activities in the protocol (Table 7, quote 2). Most parents in the XG expressed their interest in continuing to use GRP instead of conventional therapy for their child's exercise program (Table 7, quote 3). Many parents from the XG suggested to provide the GRP platform as a home-based protocol and therefore to avoid costly and time-consuming travel to the rehabilitation center (Table 7, quote 4).

**Table 7:** Parents’ suggestions to improve the protocol.

Group, parent, and ID		Quote number	Example quotes
**XG^a^**			
	Mother 22	1	“Yes, this technology-based program is helping a lot in children’s daily life. Integrating educational games, quiz games, and puzzle games would be a lot more helpful.”
	Mother 47	3	“We noticed a lot of changes during and after the computer (based) therapy program. Compared to his previous reports, we saw a lot of positive changes in his object handling and behavior. Wholeheartedly, I would request the treatment to be continued.”
	Mother 24	4	“My suggestion is that we should make these kids play these games more often, or if I could get her to do it at home, she will have more practice.”
**CG^b^**			
	Mother 7	2	“My suggestion would be to add more variety of activities to his therapy. In one session, he does a lot of activities, but it’s the same every time we come. He needs something more fun and games, something more age appropriate.”

^a^XG: experimental group.

^b^CG: control group.

## Discussion

### Principal Findings

The findings from this study establish that the parents recognized that their expectations related to improving their children's object handling and manipulation skills, including participation in ADL, were addressed during the 16-week therapy program. Parents perceived a change in their children's level of independence in their daily tasks at home, school, and leisure activities. Parents also shared the challenges they faced regarding children's participation in therapy and the experiences with the game-assisted exercise program.

Therapies such as CIMT and HABIT have established the importance of task-specific training [29]. Most parents identified the focus on handling and manipulating objects as an important feature of the GRP exercise program and that it included many different objects used in day-to-day life. Parents from the XG commented on several benefits of coupling object manipulation exercises with common computer games. Interaction with the game activities required children to manipulate each object using precise movements of varying speed and amplitudes. Different games were chosen to increase the precision level of the task (ie, small game paddles and game targets). Several studies also support the principles of goal-directed therapy for improved motor learning outcomes in children with CP [30-32].

The main focus of the game-assisted exercise program was to increase the number of repetitions. Typically, each game was played for 5-7 minutes, and the duration of each game event lasted an average of 2 seconds. Therefore, each of the objects was moved at least 100 times, which is a high number of repetitions of goal-directed activity. For most games, the game targets appear at random locations or move in unpredictable trajectories or directions (ie, variable practice). In addition, visual feedback of the game paddle was used to initiate and guide the movement responses; the child views the game events and not the object being manipulated. This type of practice would promote implicit learning of hand-eye coordination [12].

Parents from the XG reported that the exercises were challenging, yet engaging, and their children enjoyed playing the games. They felt that this gratification was important and improved the children's compliance with the therapy program. Previous studies that have compared the results of the use of computer games versus conventional therapy in terms of patient acceptability have observed similar results [33].

Many parents from the XG asked about where they could obtain other low-cost or free computer games suitable for use with the IB mouse. Understandably, updating and progressing the protocol regularly to maintain the level of difficulty and providing a new set of games to play were noted by the parents as important aspects to maintain interest and participation. Parents commented that this would likely require a large pool of different computer games. Practically, this can be difficult to achieve because, although there are a number of commercial games readily available online, not all games are suitable for each object manipulation task or for young children who have substantial motor impairments of the upper extremities.

Initially, children with severe impairments could only play games that involved slow movements and low precision (ie, large paddle size and large game target objects). On the other hand, children with moderate to mild impairment could play a larger variety of games with increased movement speeds, higher precision levels, and added cognitive content.

Parents from the CG provided mixed reviews regarding the children's interest and compliance in their exercise programs over the 16 weeks. Previous studies reported caregivers' and children's increased levels of frustration and discomfort due to the restraint used for modified CIMT and CIMT protocols [26,28-34]. Parents perceived that the cognitive activities of the GRP did contribute to improvements in their child's manual dexterity, handwriting, hand-eye coordination, and cognition, as well as notable improvement in some ADL (ie, feeding, dressing, and participation in play activities). For example, the games selected included activities for logic, problem-solving, visual search and attention, cognitive inhibition, set-shifting, verbal and nonverbal memory, color and shape recognition, and others. Previous studies have reported that the use of computer games as well as educational computer programs can benefit from academically relevant content and other cognitive skills [35]. Parents from the CG also reported that, following the supervised therapy program, their children had improved fine motor skills. They identified that one-on-one supervision provided by the therapist was important in getting the children to practice their respective exercises.

The results of the quantitative analysis (in preparation) will allow us to determine the treatment effect size and whether an exercise program using the GRP is superior to the conventional therapy program.

### Limitations

One limitation of the study was not obtaining the views of the children directly. Future studies should take into account the views and experiences of the children and not just rely upon their parents to provide this information. The number of interviews in the XG was higher than the number of interviews in the CG. It is not known why more parents in the CG declined to consent to be interviewed as compared to parents in the XG.

### Conclusion

This study demonstrated the feasibility and acceptability of the GRP platform for hand and arm function rehabilitation in children with CP. Parents who participated in the interview responded positively towards the use of the GRP and requested to continue with this therapy program after completing the 16-week intervention. Parents from the XG expressed that their children were more engaged during the GRP protocol as compared to the conventional protocols from the past. The challenges faced by parents regarding children's engagement in the protocol might be easily resolved by updating the protocol more often or by changing the difficulty levels of the tasks.

## Appendix

Multimedia Appendix 1 List of games.

Multimedia Appendix 2 CONSORT diagram.

## Abbreviations

ADL: activities of daily living
CG: control group
CIMT: Constraint Induced Movement Therapy
CP: cerebral palsy
GMFCS: Gross Motor Function Classification System
GRP: computer game–based rehabilitation platform
HABIT: hand-arm bimanual intensive training
IB: inertial based
MACS: Manual Ability Classification System
MMSE: Mini-Mental State Examination
PDMS-2: Peabody Developmental Motor Scales - Second Edition
RCT: randomized controlled trial
RTP: repetitive task practice
XG: experimental group

## References

[1] Ramey SL, DeLuca S, Stevenson RD, Case-Smith J, Darragh A, Conaway M (2019). Children with Hemiparesis Arm and Movement Project (CHAMP): protocol for a multisite comparative efficacy trial of paediatric constraint-induced movement therapy (CIMT) testing effects of dosage and type of constraint for children with hemiparetic cerebral palsy. BMJ Open.

[2] Ouyang R, Yang C, Qu Y, Koduri M, Chien C (2020). Effectiveness of hand-arm bimanual intensive training on upper extremity function in children with cerebral palsy: A systematic review. Eur J Paediatr Neurol.

[3] Jamali A, Amini M (2018). The Effects of Constraint-Induced Movement Therapy on Functions of Cerebral Palsy Children. Iran J Child Neurol.

[4] Rohrbach N, Chicklis E, Levac D (2019). What is the impact of user affect on motor learning in virtual environments after stroke? A scoping review. J Neuroeng Rehabil.

[5] Chiu H, Ada L, Lee H (2014). Upper limb training using Wii Sports Resort for children with hemiplegic cerebral palsy: a randomized, single-blind trial. Clin Rehabil.

[6] Jannink MJA, van der Wilden GJ, Navis DW, Visser G, Gussinklo J, Ijzerman M (2008). A low-cost video game applied for training of upper extremity function in children with cerebral palsy: a pilot study. Cyberpsychol Behav.

[7] Preston N, Weightman A, Gallagher J, Holt R, Clarke M, Mon-Williams M, Levesley M, Bhakta B (2016). Feasibility of school-based computer-assisted robotic gaming technology for upper limb rehabilitation of children with cerebral palsy. Disabil Rehabil Assist Technol.

[8] Gerber C, Kunz B, van Hedel HJA (2016). Preparing a neuropediatric upper limb exergame rehabilitation system for home-use: a feasibility study. J Neuroeng Rehabil.

[9] Szturm T, Polyzoi E, Marotta J, Srikesavan C (2014). An In-School-Based Program of Combined Fine Motor Exercise and Educational Activities for Children with Neurodevelopmental Disorders. Games Health J.

[10] Szturm T, Imran Z, Pooyania S, Kanitkar A, Mahana B (2021). Evaluation of a Game Based Tele Rehabilitation Platform for In-Home Therapy of Hand-Arm Function Post Stroke: Feasibility Study. PM R.

[11] Sanjay P, Kanitkar A, Szturm T, Gaonkar N, Ankolekar B (2020). A Computer Game-Assisted Repetitive Task Practice based Upper Extremity Therapy Program for Children with Spastic Unilateral Cerebral Palsy: A Single Case Study. The Indian Journal of Physiotherapy & Occupational Therapy.

[12] Maier M, Ballester BR, Verschure PFMJ (2019). Principles of Neurorehabilitation After Stroke Based on Motor Learning and Brain Plasticity Mechanisms. Front Syst Neurosci.

[13] Geerdink Y, Aarts P, Geurts AC (2013). Motor learning curve and long-term effectiveness of modified constraint-induced movement therapy in children with unilateral cerebral palsy: a randomized controlled trial. Res Dev Disabil.

[14] Muratori LM, Lamberg EM, Quinn L, Duff SV (2013). Applying principles of motor learning and control to upper extremity rehabilitation. J Hand Ther.

[15] Proctor E, Silmere H, Raghavan R, Hovmand P, Aarons G, Bunger A, Griffey R, Hensley M (2011). Outcomes for implementation research: conceptual distinctions, measurement challenges, and research agenda. Adm Policy Ment Health.

[16] Berkwits M, Inui TS (1998). Making use of qualitative research techniques. J Gen Intern Med.

[17] Kruijsen-Terpstra A, Ketelaar M, Boeije H, Jongmans M, Gorter J, Verheijden J, Lindeman E, Verschuren O (2014). Parents' experiences with physical and occupational therapy for their young child with cerebral palsy: a mixed studies review. Child Care Health Dev.

[18] Bodkin AW, Robinson C, Perales FP (2003). Reliability and validity of the gross motor function classification system for cerebral palsy. Pediatr Phys Ther.

[19] Eliasson A, Krumlinde-Sundholm L, Rösblad B, Beckung E, Arner M, Öhrvall A, Rosenbaum P (2006). The Manual Ability Classification System (MACS) for children with cerebral palsy: scale development and evidence of validity and reliability. Dev Med Child Neurol.

[20] Mutlu A, Livanelioglu A, Gunel MK (2008). Reliability of Ashworth and Modified Ashworth scales in children with spastic cerebral palsy. BMC Musculoskelet Disord.

[21] Jain M, Passi GR (2005). Assessment of a modified Mini-Mental Scale for cognitive functions in children. Indian Pediatr.

[22] van Hartingsveldt MJ, Cup EH, Oostendorp RA (2005). Reliability and validity of the fine motor scale of the Peabody Developmental Motor Scales-2. Occup Ther Int.

[23] Big Fish Games.

[24] Barzel A, Ketels G, Stark A, Tetzlaff B, Daubmann A, Wegscheider K, van den Bussche H, Scherer M (2015). Home-based constraint-induced movement therapy for patients with upper limb dysfunction after stroke (HOMECIMT): a cluster-randomised, controlled trial. The Lancet Neurology.

[25] Beveridge B, Feltracco D, Struyf J, Strauss E, Dang S, Phelan S, Wright FV, Gibson BE (2015). "You gotta try it all": Parents' Experiences with Robotic Gait Training for their Children with Cerebral Palsy. Phys Occup Ther Pediatr.

[26] Gilmore R, Ziviani J, Sakzewski L, Shields N, Boyd R (2010). A balancing act: children's experience of modified constraint-induced movement therapy. Dev Neurorehabil.

[27] Thorne S, Kirkham SR, MacDonald-Emes J (1997). Interpretive description: a noncategorical qualitative alternative for developing nursing knowledge. Res Nurs Health.

[28] Sandelowski M (1986). The problem of rigor in qualitative research. ANS Adv Nurs Sci.

[29] Gordon AM, Schneider JA, Chinnan A, Charles JR (2007). Efficacy of a hand-arm bimanual intensive therapy (HABIT) in children with hemiplegic cerebral palsy: a randomized control trial. Dev Med Child Neurol.

[30] Morgan C, Novak I, Dale RC, Guzzetta A, Badawi N (2014). GAME (Goals - Activity - Motor Enrichment): protocol of a single blind randomised controlled trial of motor training, parent education and environmental enrichment for infants at high risk of cerebral palsy. BMC Neurol.

[31] Sandlund M, Dock K, Häger Charlotte K, Waterworth EL (2012). Motion interactive video games in home training for children with cerebral palsy: parents' perceptions. Disabil Rehabil.

[32] Löwing K, Bexelius A, Brogren Carlberg E (2009). Activity focused and goal directed therapy for children with cerebral palsy--do goals make a difference?. Disabil Rehabil.

[33] Miller S, Reid D (2003). Doing play: competency, control, and expression. Cyberpsychol Behav.

[34] Mancini MC, Brandão MB, Dupin A, Drummond AF, Chagas PSC, Assis MG (2013). How do children and caregivers perceive their experience of undergoing the CIMT protocol?. Scand J Occup Ther.

[35] Blumberg F, Deater‐Deckard K, Calvert S, Flynn R, Green C, Arnold D, Brooks PJ (2019). Digital Games as a Context for Children's Cognitive Development: Research Recommendations and Policy Considerations. Soc Policy Rep.

