# Supplementary material for: Parents' Perspectives on a Computer Game–Assisted Rehabilitation Program for Manual Dexterity in Children With Cerebral Palsy: Qualitative Analysis of Expectations, Child Engagement, and Benefits
Source: JMIR Rehabil Assist Technol. 2021 May 31;8(2):e24337. doi: 10.2196/24337 (PMC8204242; doi:10.2196/24337)
Supplement: Multimedia Appendix 1 [file rehab_v8i2e24337_app1.pdf]

Appendix 1

Big Fish Games Details. Note matching and shooting games required require participants to use a small wireless optical computer mouse with left-mouse button to press when needed or small hand-held clicker with a left mouse button to press when needed.

**Big Fish Game**	**Axis Play**	**Start Difficulty**	**Type**	**Clicker**	**Precision**	**Background**	**Distractor**	**Executive Function**
Abundante	Horizontal	Difficult	Match 3	Yes	Moderate	Low Optokinetic	No	Matching and Puzzle Solving
action ball	Horizontal	Moderate	Brick Buster	No	Moderate	High Optokinetic	Yes	Visual Tracking & Spatial
Aqua Ball	Horizontal	Easy	Brick Buster	No	Low	Medium optokinetic	Yes	Visual Tracking & Spatial
Astrobugs Revenge	Horizontal	Difficult	Match 3	Yes	High	Medium optokinetic	No	Matching
Birds Town	Horizontal	Moderate	Match 3	Yes	High	Low Optokinetic	No	Matching
Brave Piglet	Vertical	Moderate	Shooting	Yes	High	Low Optokinetic	Yes	Visual Tracking & Spatial
Bricks of Egypt	Horizontal	Easy	Brick Buster	No	Variable	Low Optokinetic	Yes	Visual Tracking & Spatial

Butterfly Escape	Horizontal	Moderate	Match 3	Yes	High	Low Optokinetic	Yes	Visual Tracking & Spatial
Bubble Town	Horizontal	High	Match 3	Yes	High	Moderate Optokinetic	No	Matching, Pairing
Bubble Nauts	Horizontal	Low	Match 3	Yes	Moderate	Moderate Optokinetic	Yes	Visual Tracking & Spatial
Chicken Invaders	Variable	High	Shooting	Yes	Moderate	High Optokinetic	Yes	Search and Select
Clear It 2	Variable	High	Match 3	Yes	High	Moderate Optokinetic	No	Visual Tracking & Spatial
Egyptian Ball	Horizontal	Difficult	Brick Buster	No	Moderate	High Optokinetic	No	Search and Select
Feeding Frenzy	Variable	Difficult	Aim and Move	No	High	Low Optokinetic	Yes	Visual Tracking & Spatial
Hyperballoid 2	Horizontal	Difficult	Brick Buster	No	Moderate	High Optokinetic	No	Visual Tracking & Spatial
Invadazoid	Horizontal	Difficult	Brick Buster	No	High	Moderate Optokinetic	Yes	Visual Tracking & Spatial
Jar of Marbles	Horizontal	Easy	Match 3	Yes	Medium	Low Optokinetic	No	Matching Three , aligning

Jet Jumper	Varia ble	Difficult	Driving Game	Yes	High	High Optokinetic	Yes	Visual Tracking and Driving
Luxor 3	Horiz ontal	Moderat e	Match 3	Yes	High	Moderate Optokinetic	Yes	Match 3, Aligning
Luxor HD	Horiz ontal	Moderat e	Match 3	Yes	High	Moderate Optokinetic	Yes	Match 3, Aligning
Luxor 5th Passag e	Horiz ontal	Moderat e	Match 3	Yes	High	Moderate Optokinetic	Yes	Match 3, Aligning
Ozzy bubble s	Horiz ontal	Difficult	Aim and Move	Yes	Low	Low Optokinetic	Yes	Driving game
Ricoch et Rechar ge	Horiz ontal	Moderat e	Brick Buster	No	High	Moderate Optokinetic	No	Visual Tracking & Spatial
