# Supplementary figures and images for: Parents' Perspectives on a Computer Game–Assisted Rehabilitation Program for Manual Dexterity in Children With Cerebral Palsy: Qualitative Analysis of Expectations, Child Engagement, and Benefits
Source: JMIR Rehabil Assist Technol. 2021 May 31;8(2):e24337. doi: 10.2196/24337 (PMC8204242; doi:10.2196/24337)

## Appendix 2: CONSORT Flow Diagram

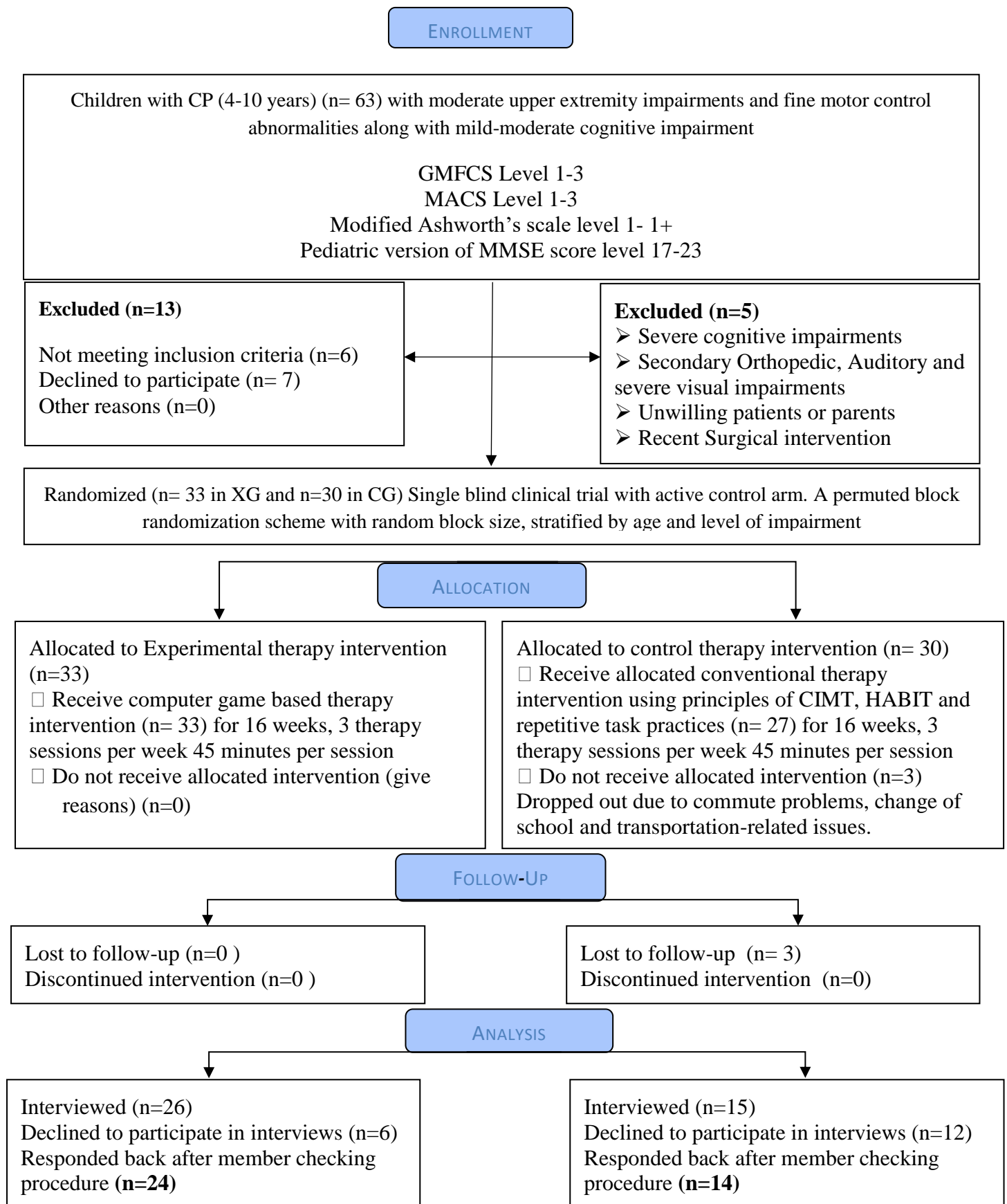

Supplement: Multimedia Appendix 2 [file rehab_v8i2e24337_app2.pdf]
